# Administration of mucuna beans (*Mucuna pruriences* (L.) DC. *var. utilis*) improves cognition and neuropathology of 3 × Tg-AD mice

**DOI:** 10.1038/s41598-022-04777-z

**Published:** 2022-01-19

**Authors:** Fumiko Konishi, Tadasu Furusho, Yoshiyuki Soeda, Jun Yamauchi, Shoko Kobayashi, Masatoshi Ito, Takuma Araki, Sarasa Kogure, Akihiko Takashima, Susumu Takekoshi

**Affiliations:** 1 Department of Nutrition, Kagawa Nutrition University, 3-9-21 Chiyoda, Sakado, Saitama 350-0288 Japan; 2 Department of International Food and Agricultural Science, Tokyo University of Agriculture, 1-1-1 Sakuragaoka, Setagaya-ku, Tokyo, 156-8502 Japan; 3 Laboratory for Alzheimer’s Disease, Department of Life Sciences, Faculty of Science, Gakushuin University, 1-5-1 Mejiro, Toshima-ku, Tokyo, 171-8588 Japan; 4 Department of Applied Biological Chemistry, Graduate School of Agricultural and Life Sciences, The University of Tokyo, Yayoi, Bunkyo-ku, Tokyo, 113-8657 Japan; 5 Support Center for Medical Research and Education, Tokai University, 143 Shimokasuya, Isehara, Kanagawa 259-1193 Japan; 6 Department of Ecological Symbiotic Science, Graduate School of Agriculture, Tokyo University of Agriculture, 1-1-1 Sakuragaoka, Setagaya-ku, Tokyo, 156-8502 Japan; 7 Department of Cell Biology, Division of Host Defense Mechanism, Tokai University School of Medicine, 143 Shimokasuya, Isehara, Kanagawa 259-1193 Japan

**Keywords:** Disease prevention, Neurological disorders

## Abstract

Alzheimer’s disease (AD) is a neurodegenerative disorder characterized by the accumulation of extracellular amyloid-beta peptides (Aβ) resulting in senile plaques and intracellular hyperphosphorylated tau protein resulting in neurofibrillary tangles (NFTs). Mucuna beans (*Mucuna pruriences* (L.) DC. *var. utilis*) are unique plants containing 3–9% L-3,4-dihydroxyphenylalanine (L-DOPA). Here we investigated the effect of the administration of Mucuna beans on AD prevention by feeding triple-transgenic mice (3 × Tg-AD mice) with a diet containing Mucuna beans for 13 months. The levels of Aβ oligomers and detergent-insoluble phosphorylated tau decreased in the brain of mice fed with Mucuna beans (Mucuna group) compared to those of the Control group. Aβ accumulation and phosphorylated tau accumulation in the brain in the Mucuna group were also reduced. In addition, administration of Mucuna beans improved cognitive function. These results suggest that administration of Mucuna beans may have a preventive effect on AD development in 3 × Tg-AD mice.

## Introduction

Alzheimer’s disease (AD) is the most common type of dementia accompanied by memory impairment and neuronal loss. The pathological features of AD are the accumulation of aggregated amyloid-beta peptides (Aβ) and neurofibrillary tangles (NFTs) composed of hyper-phosphorylated tau protein^1^.

Aβ molecules have self-aggregation properties and are prone to forming soluble oligomers with a β-sheet structure, protofibrils, and then insoluble fibrils as the final stage^2,3^. A growing body of studies has suggested that soluble Aβ oligomers are neurotoxic and potently inhibit long-term potentiation^4–6^. Shankar et al*.* found that Aβ oligomers disrupted synaptic function as well as memory function^4^. They also indicated that Aβ dimers are the smallest neurotoxic molecules^4^.

Tau proteins are primarily present in the distal portion of axons and control microtubule stabilization, regulate axonal transport, and maintain neuronal function^7^. When tau is abnormally phosphorylated in its microtubule-binding domain, it loses the ability to bind and stabilize microtubule assembly, leading to the dysfunction of axonal transport, synaptic loss, and neuronal death^7^. Dislodged tau from microtubules self-assembles into tau oligomers (Sarkosyl-soluble), granular tau oligomers (Sarkosyl-insoluble), and fibrils that form NFTs^8^. Granular tau oligomers are a major neurotoxic protein and are associated with neuronal loss^9,10^. Taken together, the formation of neurotoxic oligomers seems to be a common characteristic for Aβ and tau. Therefore, it might be an attractive strategy for AD prevention to search for compounds that inhibit the aggregation of both Aβ and tau.

Hamaguchi et al. found that feeding AD-model mice with a diet containing Rosmarinic acid (RA) reduced Aβ oligomer formation^11^. Regarding the mechanism of this reduction, Hase et al*.* demonstrated that feeding with a RA-containing diet increased the levels of catecholamines including L-3,4-dihydroxyphenylalanine (L-DOPA), norepinephrine (NE), dopamine (DA), and 3,4-dihydroxyphenylacetic acid (DOPAC) in the mouse brain, through downregulation of the expression of DA-degrading enzymes^12^. Moreover, these catecholamines inhibited Aβ aggregation in vitro. Therefore, an increase in catecholamine levels in the brain might be beneficial for AD pathogenesis.

Compounds with a catechol skeleton including L-DOPA, DA, and NE also have an inhibitory effect against the tau aggregation in a dose-dependent manner in vitro^13^. The catechol moiety plays an inhibitory role in tau aggregation by interacting with Cys residues in tau^13^. Therefore, compounds with a catechol skeleton structure, including catecholamines are expected to be an agent for preventing AD by hindering the aggregation of both Aβ and tau.

The Mucuna bean (*Mucuna pruriences* (L.) DC. *var. utilis*) plant is a heavy cropper, and these beans are eaten mainly in Asia and Africa^14^. Mucuna beans contain 3–9% L-DOPA, while other plants rarely contain such high amount of the molecule^14,15^. L-DOPA is potentially toxic when ingested in large doses. Thus, intake of Mucuna beans in large amounts at once often causes nausea and vomiting in humans owing to their high L-DOPA content^16^. However, if Mucuna beans are processed properly to control the amount of L-DOPA, the intake of Mucuna beans might be effective for the prevention of AD through inhibition of Aβ and tau aggregation.

To investigate this possibility, we examined the biological effect of Mucuna-bean administration on AD prevention by feeding triple-transgenic mice (3 × Tg-AD mice) that express three mutant human transgenes: amyloid precursor protein (APP_swe_), presenilin-1 (PS1_M146V_), and four-repeat tau (tau_P301L_). These mice progressively develop AD pathology, including Aβ accumulation and phosphorylated tau accumulation^17^. 3 × Tg-AD mice also develop age-dependent cognitive impairment before the accumulation of Aβ and phosphorylated tau with aging^18^. Mucuna beans were fed to these mice, and the levels of Aβ oligomers and phosphorylated tau in the brain were compared to mice fed with a standard diet. Behavioral changes and accumulation of Aβ and phosphorylated tau in the brain were also assessed.

## Materials and methods

The present study is reported in accordance with ARRIVE guidelines.

### Animals and treatment

Four-week-old female 3 × Tg-AD mice harboring three mutant genes (APPswe, PS1 _M146V_, and tau _P301L_)^17^ were purchased from the Jackson Laboratory, Bar Harbor, ME, USA. The mice were housed individually at a temperature of 24 °C ± 2 °C, 40%–50% relative humidity, and a 12 h light–dark cycle with ad libitum access to sterilized water. Starting at 1 month of age, the mice were divided into two groups: the Control group was fed with a Control diet (AIN-76 diet)^19^ (n = 9), and the Mucuna group was fed with a diet containing Mucuna-bean powder (Mucuna diet, n = 9) for 13 months. The Mucuna-bean powder used for preparing the Mucuna diet was processed as follows: Mucuna beans (whose chemical composition is shown in the Table S1), were of a gift from a farmer who had been cultivating this bean in Kumamoto prefecture in Japan. The written consent of the beans to be used for this research has been obtained. The use of the Mucuna beans in the present study complies applicable with international, national and /or institutional guidelines. The Mucuna beans were soaked in distilled water for 24 h and then autoclaved at 121 °C for 40 min, lyophilized, and milled (New Power Mill, Osaka Chemical Co., Ltd., Japan). The Mucuna-bean powder contained 2.88% L-DOPA. The daily intake of L-DOPA derived from Mucuna-bean powder was adjusted to 2 mg/kg body weight/day per mouse throughout the feeding period. The Mucuna-bean powder content in the Mucuna diet varied from 0.028 to 0.091% in accordance with body weight. Body weight was monitored weekly. At the age of 14 months, mice were sacrificed under anesthesia with 200 mg/kg body weight of sodium pentobarbital delivered intraperitoneally, and the brain of each mouse was quickly harvested and sagittally bisected. The left hemisphere was fixed in 4% paraformaldehyde overnight for histological studies. The right hemisphere was dissected to isolate the striatum, hippocampus, and cerebral cortex and was stored at − 80 °C for biochemical analyses.

All experiments reported herein were approved by the Animal Care Advisory Committee of Kagawa Nutrition University and were performed in accordance with the relevant guidelines and regulations (Permit Number: 18–2).

### Behavioral test: Y-maze test

The Y-maze apparatus consisted of three arms (38 cm long, 12.5 cm wide, and 12.5 cm deep; Sanki Kagaku Kogei, Japan). Each mouse, at the age of 12 months, was placed at the center of the maze and allowed to freely explore the maze for 5 min. An entry into an arm was considered complete when all four limbs were within the arm. An alteration was defined as three consecutive entries into three different arms (A, B, C or B, C, A, etc.)^20^. The percentage alteration score was calculated as follows: the total alteration number/(total number of entries − 2) × 100.

### Preparation of recombinant Tau protein

Recombinant human tau protein (2N4R) cDNA in pET vectors was expressed in BL21 (DE3) *Escherichia coli* cells and purified as previously reported^13^. After *E. coli* expressing tau was sonicated and boiled, recombinant tau proteins in the heat-stable fraction were purified using ion-exchange chromatography (Cellufine Phosphate; JNC Corp.), ammonium sulfate fractionation, gel filtration chromatography (NAP10 column; GE Healthcare), and reverse-phase HPLC (COSMOSIL Protein-R Waters; Nacalai Tesque Inc.). After freeze-drying, recombinant tau proteins were dissolved in Milli-Q water and stored at − 80 °C.

### Thioflavin T assay

Mucuna-bean extracts for the Thioflavin T (ThT) assay were prepared as follows: Mucuna beans were incubated in distilled water at 100 °C for 4 h and boiled for 1 h. Boiled Mucuna beans were homogenized in 0.4 M phosphate buffer (pH = 4.0) and centrifuged (9,000 rpm, 10 min, 4 °C). The supernatant was filtered through 0.45 μm filters and subjected to a ThT assay. The ThT assay for Aβ was performed as previously reported^21^. Mucuna-bean extracts (Mucuna-bean concentration of 0.273 mg/ml or 2.73 mg/ml which corresponds to10 μM L-DOPA or 100 μM L-DOPA, respectively), Aβ_1-42_ (10 μM, PEPTIDE INSTITUTE, INC., Osaka, Japan), and ThT (10 μM) were mixed in PBS and incubated at 37 °C. The ThT fluorescence intensity was recorded with an Ex/Em = 450 nm/492 nm using a microplate-reader fluorometer (Corona Electric, Japan). The ThT assay for tau was also performed as previously reported^13^. Recombinant wild-type 2N4R tau (10 μM), Mucuna-bean extracts (Mucuna-bean concentration of 0.273 mg/ml or 2.73 mg/ml which corresponds to10 μM L-DOPA or 100 μM L-DOPA, respectively), and ThT (10 μM) were mixed in HEPES buffer (10 mM HEPES, pH = 7.4; 100 mM NaCl) and incubated with heparin (0.06 mg/ml; Acros Organics) at 37 °C. The ThT fluorescence intensity was recorded every hour with an Ex/Em = 420 nm/500 nm using a multiplate-reader fluorometer (1420 ARVO MX, Perkin Elmer). All samples were tested in triplicate.

### L-DOPA analyses

The L-DOPA content of Mucuna-bean extracts or powder was analyzed using a reversed-phase column WAKOSIL II 5C18 RS (Φ4.6 × 250 mm). This column was placed in a JASCO CO-2067 Plus HPLC column oven and analysis was conducted at 40 °C. The mobile phase was phosphate buffer (pH = 2) / methanol (90:10) with a flow rate of 1 mL/min. The eluate was monitored at 200 nm.

### Tissue extraction for biochemical studies

Tissue extraction was performed as previously described^13^. Frozen brain tissues (striatum, hippocampus, and cerebral cortex) were homogenized in 7.5 volumes of TBS buffer containing 50 mM Tris (pH = 7.4), 150 mM NaCl, 1 mM EDTA, 1 mM EGTA, protease inhibitors, and phosphatase inhibitors. The homogenates were centrifuged (23,000 rpm, 15 min, 4 °C) in a TLA-55 rotor (Beckman Coulter) and separated into the supernatant (TBS-soluble fraction) and pellet. The pellets were resuspended in 0.32 M sucrose containing 10 mM Tris (pH = 7.4), 0.8 M NaCl, and 1 mM EGTA and were then centrifuged (23,000 rpm, 15 min, 4 °C) in a TLA-55 rotor. Supernatants were collected and treated with 1% Sarkosyl for 1 h at 37 °C. They were then centrifuged (60,000 rpm, 1 h, 4 °C) in a TLA-110 rotor and separated into the supernatant and pellet (Sarkosyl-insoluble fraction). Samples from TBS-soluble and Sarkosyl-insoluble fractions were dissolved in Laemmli SB including 2-mercaptoethanol and were then boiled for 5 min.

### Immunoblotting analysis

Immunoblotting analysis was performed as previously described^22^. The protein concentration of the TBS-soluble and Sarkosyl-insoluble fractions was determined using a DC protein assay kit (Bio-Rad, Hercules, CA, USA). Fifty-microgram protein from the TBS-soluble fraction was applied to NOVEX 10%–20% Tricine gel (Thermo Fisher). The separated proteins were transferred to nitrocellulose membranes (Millipore, Billerica, MA, USA) and were subjected to heat treatment (boiling in PBS, 15 min). After blocking for 60 min at room temperature with 2.5% skim milk in PBS containing 0.05% Tween 20, the membranes were incubated for 1 h at room temperature with primary antibody (6E10, 1:1000, BioLegend). After washing the membrane with PBS containing 0.05% Tween 20, the blots were incubated with horseradish peroxidase-linked secondary antibodies at room temperature. Immune complexes were visualized by Western Lightning Ultra (Perkin Elmer).

Thirty-microgram protein from the Sarkosyl-insoluble fraction was applied to NOVEX 5–20% e-Pagel (ATTO). The separated proteins were transferred to nitrocellulose membranes (Millipore). After blocking for 1 h at room temperature with 2.5% skim milk in PBS containing 0.05% Tween 20, the membranes were incubated overnight at 4 °C with primary antibody (AT8, 1:1000, Thermo Fisher Scientific). After washing the membrane with PBS containing 0.05% Tween 20, the blots were incubated with horseradish peroxidase-linked secondary antibodies (anti-mouse IgG, GE Healthcare) at room temperature. Immune complexes were visualized by Western Lightning Ultra (Perkin Elmer). The results were analyzed by densitometry (NIH ImageJ-Fiji, v-1.45 software) and expressed as percentage of control values.

### Immunohistochemistry

Immunohistochemical analysis was performed as previously described^22^. The left hemisphere was fixed in 4% paraformaldehyde overnight. Paraffin-embedded sections (sectioned in the coronal plate at 4 μm thick) were deparaffinized. For antigen retrieval, the sections for Aβ were treated with proteinase K (DAKO, S3004) for 10 min and then treated with 90% formic acid for 5 min. The sections for phosphorylated tau were treated with Target Retrieval Solution (pH 9.0; DAKO, S2367) at 120 °C for 10 min. Then, both sections were immersed in 0.3% hydrogen peroxide in methanol for 10 min. After treating with 5% normal rabbit serum for 10 min, the sections were incubated overnight at 4 °C with primary antibodies that were pre-mixed with secondary antibodies (anti-mouse IgG2b, goat Fab) at room temperature for 20 min. Primary antibodies for Aβ and phosphorylated tau were 4G8 (1:1000, BioLegend) and AT8 (1:1000, Thermo Fisher Scientific), respectively. The secondary antibodies were visualized using a HistoFine kit (Nichirei, Japan), followed by a 3,3′-diaminobenzidine reaction. Finally, the sections were counterstained with hematoxylin and observed using an Olympus BX63 microscope equipped with an Olympus DP73 digital camera. Consecutive sections were incubated in the absence of primary antibodies to ensure the specificity of staining.

Quantification of the 4G8-positive Aβ area and the AT8-positive tau area in the brain was performed using ImageJ-Fiji (v-1.45 NIH) after adjusting for the threshold, and the results were expressed as percentage of control values.

### Statistical analysis

Values are expressed as means ± SEM. Differences were analyzed using Student’s *t*-test, Welch’s *t*-test, or one-way analysis of variance followed by Tukey’s multiple comparisons test. Statistical significance was considered when *p* < 0.05.

## Results

### Mouse characteristics

**Figure 1:**
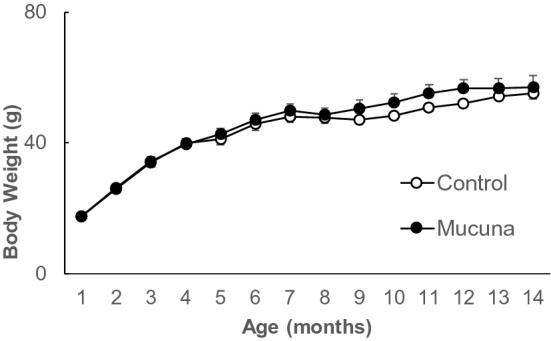
Monitoring the body weight of Mucuna treated mice.

During this experiment, the Control group mice and the Mucuna group mice received a standard diet and a diet containing Mucuna-bean powder, respectively, and body weight was determined weekly. As shown in Fig. 1, body weight was not significantly different between the groups during the 13-month treatment.

All mice were weighed weekly from the beginning until the end of the study. Values are means ± SEM. At the age of 1 month; Control n = 9, Mucuna n = 9, at the age of 14 months; Control n = 8, Mucuna n = 7.

### Inhibition of Aβ aggregation and tau aggregation by Mucuna-bean extracts

The effect of Mucuna-bean extracts on Aβ aggregation and tau aggregation in vitro was examined using the ThT assay. This assay is based on monitoring the fluorescence change of ThT when binding to aggregated Aβ and tau^23^. The intensity of fluorescence was determined in the presence of Mucuna-bean extracts (Mucuna-bean concentration of 0.273 mg/ml or 2.73 mg/ml which corresponds to10 μM L-DOPA or 100 μM L-DOPA, respectively).

**Figure 2:**
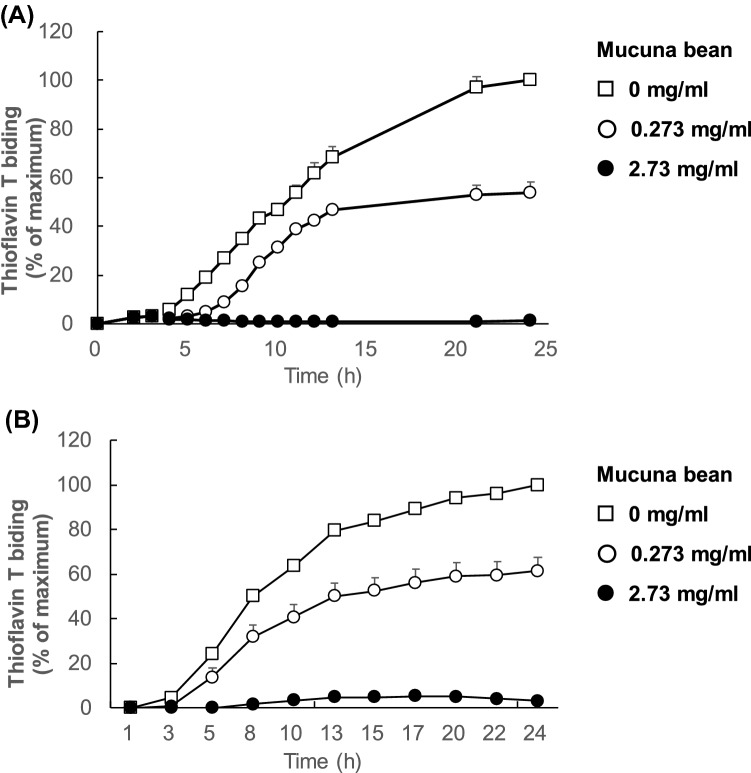
Inhibitory effect of Mucuna-bean extracts on Aβ aggregation and tau aggregation. (**A**) Mucuna-bean extracts dose-dependently inhibited Aβ aggregation at 9–25 h (*p* < 0.05). Results are expressed as percentage of maximum fluorescence (means ± SEM of triplicate experiments; n = 3). (**B**) Mucuna-bean extracts dose-dependently inhibited tau aggregation at 5–24 h (*p* < 0.05). Results are expressed as percentage of maximum fluorescence (means ± SEM of triplicate experiments; n = 3). Mucuna- bean concentration: □: 0 mg/ml, ○: 0.273 mg/ml, ●: 2.73 mg/ml.

When Mucuna-bean extracts were added to Aβ_1-42_, the fluorescence intensity was suppressed in a dose-dependent manner (Fig. 2A). A dose-dependent reduction was also observed for heparin-induced ThT fluorescence when Mucuna-bean extracts (Mucuna-bean concentration of 0.273 mg/ml or 2.73 mg/ml which corresponds to10 μM L-DOPA or 100 μM L-DOPA, respectively) added to recombinant wild-type-2N4R tau (Fig. 2B). These results indicate that Mucuna-bean extracts had an inhibitory effect on both Aβ and tau aggregation in a dose-dependent manner.

### Immunoblotting analysis of Aβ and tau

**Figure 3:**
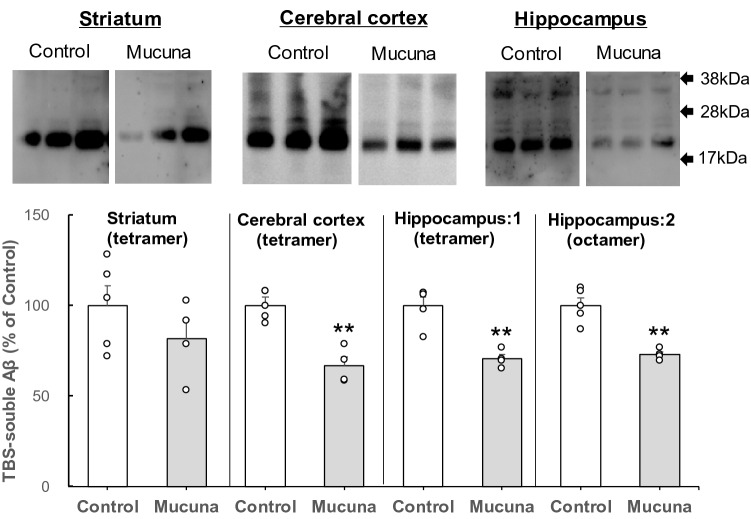
Immunoblotting analysis of Aβ oligomers in the TBS-soluble fraction.

To investigate the effect of a Mucuna diet on Aβ and tau aggregation in vivo, 3 × Tg-AD mice were fed with a Mucuna diet for 13 months, starting at 1 month of age. After sacrificing, the striatum, hippocampus, and cerebral cortex were collected and homogenized. Homogenates were separated into a TBS-soluble fraction and a Sarkosyl-insoluble fraction. TBS-soluble fractions were subjected to immunoblotting analysis of Aβ_1-42_. Significantly reduced levels of Aβ_1-42_ tetramers and Aβ_1-42_ octamers were observed in the hippocampi (*p* < 0.01) of the Mucuna group compared to those of the Control group (Fig. 3). Significantly reduced levels of Aβ_1-42_ tetramers were also observed in the cerebral cortexes (*p* < 0.01) of the Mucuna group compared to those of the Control group (Fig. 3). However, levels of Aβ_1-42_ tetramers in the striatum of the Mucuna group were not significantly different from those of the Control group. Aβ_1-42_ octamers were not detected in the cerebral cortex and striatum.

TBS-soluble fractions were obtained from the striatum, cerebral cortex, and hippocampus homogenates from 3 × Tg-AD mice fed with the Mucuna diet (Mucuna group) or the Control diet (Control group). Fifty-microgram protein from the TBS-soluble fraction was applied to NOVEX 10%–20% Tricine gel (Thermo Fisher). The arrows refer to the protein ladders. Levels of TBS-soluble Aβ oligomers were analyzed by immunoblotting using 6E10 antibodies and quantified. Hippocampus:1 shows Aβ_1-42_ tetramers; Hippocampus:2 shows Aβ_1-42_ octamers. Densitometry of Aβ oligomer immunoreactivity was quantified. Results are shown as percentage of control values. Values represent means ± SEM, each point represents the individual data of the mice. **p < 0.01 versus control by Student's *t*-test. n =4.

**Figure 4:**
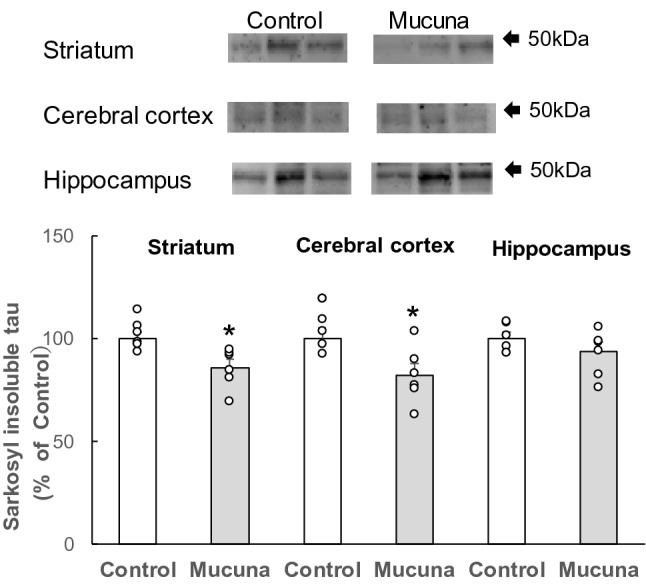
Immunoblotting analysis of phosphorylated tau in the Sarkosyl-insoluble fraction.

Sarkosyl-insoluble fractions were subjected to immunoblotting analysis of phosphorylated tau. Phosphorylated tau levels were found to be significantly reduced in the striatum (*p* < 0.05) and cerebral cortex (*p* < 0.05) from the Mucuna group as compared to those from the Control group (Fig. 4). In the hippocampi of the Mucuna group, the phosphorylated tau levels did not reach statistical significance. These results indicate that Aβ and tau aggregation were suppressed in the Mucuna group.

The Sarkosyl-insoluble fractions were obtained from the striatum, cerebral cortex, and hippocampus homogenates of 3 × Tg-AD mice fed with the Mucuna diet (Mucuna group) or the Control diet (Control group). Thirty-microgram protein from the Sarkosyl-insoluble fraction was applied to NOVEX 5%–20% e-Pagel (ATTO). The arrow refers to the protein ladders. Levels of phosphorylated tau in the Sarkosyl-insoluble fraction were analyzed by immunoblotting using AT8 antibodies. Densitometry of phosphorylated tau immunoreactivity was quantified. Results are shown as percentage of control values. Values represent means ± SEM, each point represents the individual data of the mice. *p < 0.05 versus control by Student's *t*-test. n = 6.

### Immunohistochemistry of Aβ and phosphorylated tau accumulation

**Figure 5:**
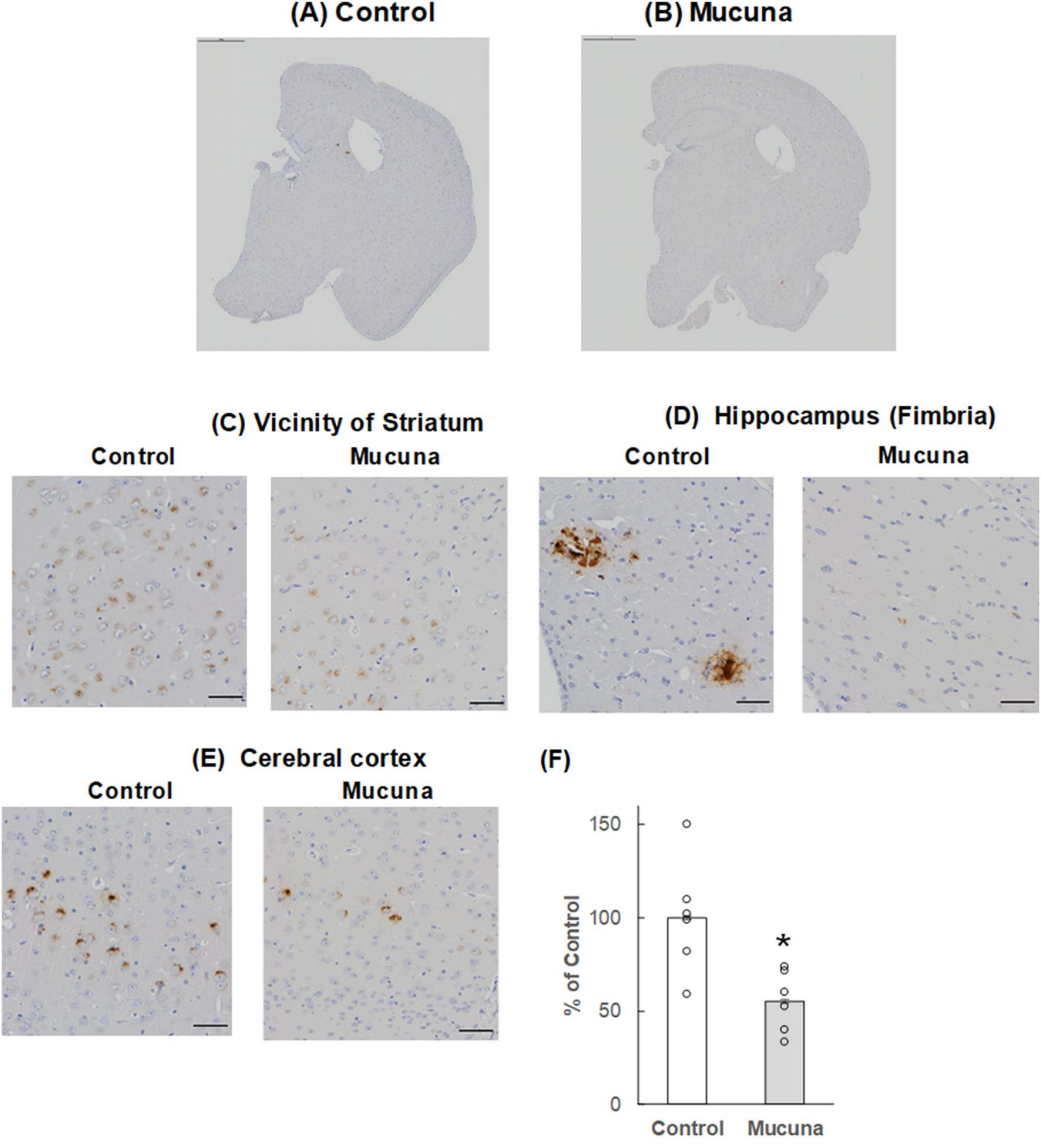
Aβ accumulation in the brain. (**A**–**E**): Representative images of the 4G8-positive Aβ accumulation immunostained with 4G8 antibody in brain sections. (**A**) hemisphere of the Control mouse, (**B**) hemisphere of the Mucuna mouse, (**C**) Vicinity of Striatum. (**D**) Hippocampus (Fimbria). (**E**) Cerebral cortex. The scale bars in (**A**,**B**) represent 1000 μm, and those in (**C**–**E**) represent 50 μm. (**F**) Percentage of the 4G8-positive Aβ area in the brain. Results are shown as percentage of control values. Values represent means ± SEM, each point represents the individual data of the mice. *p < 0.05 versus control by Student's *t*-test. n = 6.

**Figure 6:**
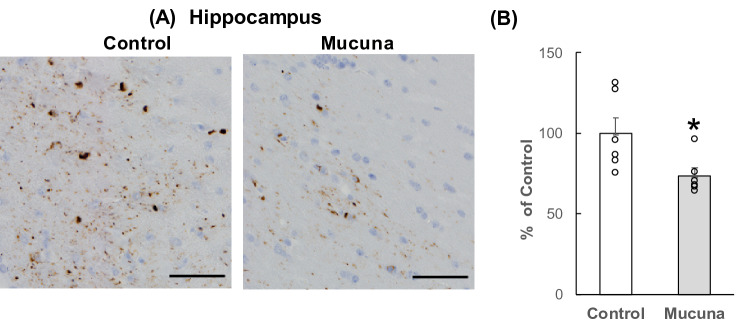
Phosphorylated tau accumulation in the brain. (**A**) Representative images of the AT8-positive tau accumulation immunostained with AT8 antibody. AT8 antibody-positive phosphorylated tau accumulation was mainly observed in the hippocampus (fimbria). The scale bar represents 50 μm. (**B**) Percentage of the AT8-positive tau area in the brain. Results are shown as percentage of control values. Values represent means ± SEM, each point represents the individual data of the mice. *p < 0.05 versus control by Student's *t*-test. n = 6.

To test whether a Mucuna diet affects Aβ and phosphorylated tau accumulation in the brain, immunohistochemical analysis was performed. 4G8 antibody-positive Aβ accumulation was observed in the vicinity of the striatum, the cerebral cortex, and the fimbria that is the nerve fiber bundle along the medial edge of the hippocampus, plays a role in controlling spatial working memory^24^. Aβ accumulation in the Mucuna group was significantly lower than that of the Control group (Fig. 5, *p* < 0.05). A significant reduction of AT8 antibody-positive phosphorylated tau accumulation was mainly observed in the fimbria. Phosphorylated tau accumulation in the Mucuna group was significantly lower than that of the Control group (Fig. 6, *p* < 0.05). These data indicate that Aβ and phosphorylated tau accumulation in the brain was suppressed in the Mucuna group.

### Behavioral test: Y-maze

**Figure 7:**
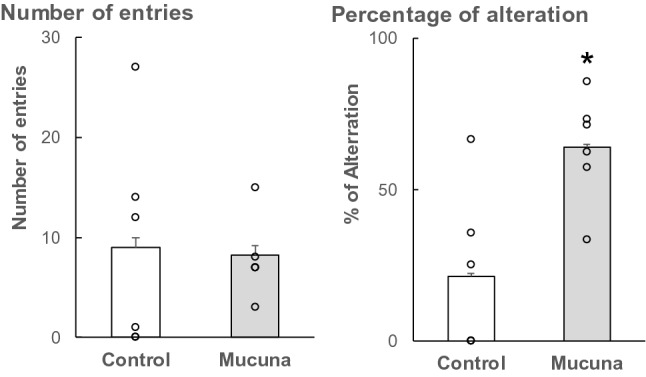
Y-maze performance. The mice were tested with the Y-maze paradigm for the number of entries and the percentage of alteration. Values represent means ± SEM, Values represent means ± SEM, each point represents the individual data of the mice. *p < 0.05 versus control by Welch's *t*-test. n = 6.

To determine the effect of the Mucuna diet on spatial working memory, mice were tested using a Y-maze apparatus at 12 months of age. The Y-maze test is a behavioral test to evaluate the spatial working memory (as measured by the percentage of alteration) and the locomotor activity (as measured by the number of entries)^20^. There were no significant differences in the number of entries between the Control group and the Mucuna group. However, the percentage of alteration was higher in the Mucuna group than in the Control group (Fig. 7, *p* < 0.05). These data suggest that administration of Mucuna beans improved behavioral performance related to spatial working memory without changes in locomotor activity.

## Discussion

The present study was conducted to investigate the biological effect of Mucuna-bean administration on AD prevention in 3 × Tg-AD mice. Since Mucuna beans contain 3%–9% of L-DOPA, a large intake of Mucuna beans causes gastrointestinal disturbances, including vomiting^16^. Therefore, in the present study, the dose level of L-DOPA in the Mucuna diet was adjusted to 2 mg/kg weight/day during the feeding period. The growth curve of the Mucuna group was nearly the same as that of the Control group over the treatment period. This result implies that the dose level of L-DOPA was appropriate. In our preliminary study, the effect of dietary supplementation of Mucuna beans on the mouse growth curve and memory function was evaluated using SAMPR1 (Senescence-Accelerated Mouse Resistant 1, Control) and SAMP8 (Senescence-Accelerated Mouse Prone 8) mice. Results indicated that the Mucuna beans supplementation does not alter the mice’s growth curves, or memory function, suggesting that wild type mice are irresponsive to dietary supplementation of Mucuna beans.

Various polyphenols derived from foods, including quercetin, RA, myricetin, and epigallocatechin gallate, have been reported to inhibit Aβ aggregation in vitro^25,26^. The phenolic hydroxyl groups of polyphenols, especially the catechol skeleton (1,2-dihydroxybenzene), are essential for the inhibition of Aβ aggregation in vitro^27^. Polyphenols with a catechol skeleton undergo autoxidation, generating *o-*quinones, which react with nucleophilic amino acid residues , including Cys (sulfhydryl groups; SH) and Lys (free amino groups; NH_2_)^28^. Generated *o-*quinones covalently bind to the Lys16 and Lys28 residues of Aβ_1-42_, resulting in destabilization of Aβ_1-42_ aggregates^27^. Meanwhile, using ThT assay, recent studies have shown that L-DOPA, which also has a catechol skeleton, inhibits Aβ_1-42_ aggregation in vitro^12,29^. In the present study, Mucuna-bean extracts inhibited Aβ aggregation dose-dependently in vitro (Fig. 2A). This result suggests that the L-DOPA in Mucuna-bean extracts might be autoxidized to dopaquinones and bind to Aβ_1-42_, resulting in the inhibition of Aβ_1-42_ aggregation.

It has been reported that monoamines with a catechol skeleton (1,2-dihydroxybenzene) including L-DOPA, DA, NE, and epinephrine inhibit tau aggregation in vitro^13^. However, compounds with a 1-hydroxybenzene skeleton, including octopamine and 3-methoxytyramine did not inhibit tau aggregation^13^. Therefore, the catechol skeleton plays an important role in the inhibition of tau aggregation. The underlying mechanism for the inhibition of tau aggregation is thought to be as follows: compounds with a catechol skeleton bind and interact with Cys residues of tau and prevent disulfide bonding between Cys residues in tau^13^. In the present study, Mucuna-bean extracts also dose-dependently inhibited tau aggregation (Fig. 2B). Thus, it is likely that L-DOPA in Mucuna-bean extracts might contribute to the inhibition of tau aggregation through interaction with the Cys residues of tau.

In the mice treated with Mucuna diet, a significant decrease in Aβ_1-42_ tetramer and octamer levels in the hippocampus was detected by immunoblotting analysis in the TBS-soluble fraction (Fig. 3). Octamers are formed by two tetramers facing each other^30^. Soluble Aβ oligomers play a role in initiating cognitive impairment^2,31,32^. For example, repeated hippocampal injections of low-order Aβ_1-42_ oligomers in the brain of awake mice caused neuronal loss, tau hyperphosphorylation, and memory deficits^33,34^. Low-order Aβ oligomers have the basic structure of protofibrils, and their neurotoxicity becomes more severe with oligomer order^35^. Regarding the mechanism underlying neurotoxicity of Aβ oligomers, several hypotheses have been proposed. For example, Aβ oligomers form ion channels when embedded into the cell membrane to cause an abnormal ion flux through the membrane, inducing cell death^36–38^. Recently, Ciudad et al. demonstrated that Aβ_1-42_ tetramers and octamers form pores within the membrane mimicking the lipid bilayer, which lead to membrane disruption by allowing water to permeate^30^. These results suggest that a diet containing Mucuna beans might contribute to the maintenance of neuronal membrane integrity by reducing the Aβ_1-42_ tetramer and octamer levels.

6E10 antibody was used to detect Aβ oligomers in the immunoblotting analysis. However, it is difficult to determine whether the Mucuna bean effect is due to the reduction of Aβ (clearance), or APP (amyloid precursor protein) processing (Aβ production). Therefore, it is of interest to clarify if the Aβ lowering effect of Mucuna beans is due to either APP processing, or the reduction of Aβ. Although we were unable to clarify this mechanism further due to the limited amounts of brain samples, it would be valuable to determine changes of APP levels.

In the present study, immunoblotting analysis showed that the levels of phosphorylated tau in the Sarkosyl-insoluble fraction were lower in the brain of mice from the Mucuna group. Phosphorylated tau in the Sarkosyl-insoluble fraction is neurotoxic and potently linked to neuronal loss in the brain of AD model mouse^39^. It has been reported that isoproterenol, an adrenergic receptor agonist that has a catechol skeleton structure, inhibits tau aggregation in vitro. Moreover, administration of isoproterenol reduced the levels of phosphorylated tau in the Sarkosyl-insoluble fraction and neuronal loss in the brain of P301L tau-transgenic mice^13^. The catechol skeleton of isoproterenol is responsible for inhibiting tau aggregation through the interaction with Cys residues of tau^13^. These results suggest that L-DOPA in Mucuna beans might contribute to the reduction in phosphorylated tau levels in the Sarkosyl-insoluble fraction via inhibition of tau aggregation in the brain of mice from the Mucuna group.

In the present paper, AT8 antibody, which can detect both pS202 and pT205, was used to detect phosphorylated tau. However, it would be ideal to confirm the results using other types of anti-phospho-tau antibodies such as pS199, pT205, pS396, pS404, or pS422.

Regarding total tau protein levels, no significant difference was observed in the hole brain homogenates from either the Mucuna group or the Control group (data not shown).

In the present study, soluble phosphorylated tau levels in the TBS-soluble fraction were not measured. Instead, phosphorylated tau in the Sarkosyl-insoluble fraction was focused on for the following reasons: it has been reported that administration of isoproterenol did not alter phosphorylated tau levels in the TBS-soluble fraction^13^ and phosphorylated tau in the Sarkosyl-insoluble fraction is reported to cause major neurotoxicity^9,10^.

Regarding the externally administered L-DOPA metabolism, it is shown to be metabolized to monoamines, such as DA and DOPAC. The concentration ratio of monoamines in the cerebral cortex of the mouse treated with RA was approximately 100:10:30:1 DA: DOPAC: NE: L-DOPA^12^. It has been reported that 3xTg-AD mice show an age-related decline in cognitive function and dopamine release in the insular cortex as well as significant memory impairment at the age of 10 months, but that 5- and 10-month-old wild type mice and 5-month-old 3×Tg-AD mice do not^40^. However, cortical administration of a dopamine reuptake blocker increased dopamine levels and attenuated memory impairment in 10-month-old 3×Tg-AD mice^40^. Yadav et al*.* treated Parkinson’s disease (PD) model mice with Mucuna beans and found that the levels of DA and DOPAC increased in the nigrostriatal region^41^. These studies suggest that the reduction of Aβ oligomers and phosphorylated tau in 3×Tg-AD mice fed with a Mucuna diet is presumably due to an activation of dopaminergic neuron by the increase of Mucuna beans derived monoamines, including DA and DOPAC.

Aβ accumulation in the brain of 3 × Tg-AD mice begins to appear in the cortex at the age of 6 months and progresses to the hippocampus^17,18^. Phosphorylated tau accumulation appears in the hippocampus at the age of 12 months and progresses to the cerebral cortex^17,18^. Injection of Aβ oligomers to the brain of AD-model mice has been reported to promote AD pathology, including Aβ accumulation^33^. Conversely, a reduction of Aβ accumulation was observed in AD-model mice receiving passive immunization with anti-oligomeric antibodies^42^. In the present study, immunoblotting analysis showed a reduction in Aβ_1-42_ tetramer and octamer levels in the brain of mice from the Mucuna group. Therefore, suppression of Aβ accumulation in the brain of these mice might be affected by the reduction in Aβ_1-42_ tetramer and octamer levels.

To evaluate the spatial working memory, the Y-maze test was performed^20^. Lauretti et al*.* reported that the number of entries of 3 × Tg-AD mice gradually decreases as they become older and is reduced to below 10 at the age of 9–12 months in the Y-maze test^43^. Our Y-maze test was performed at the age of 12 months with about 8–9 entries. In the present study, mice in the Mucuna group improved behavioral performance related to spatial working memory compared with those in the Control group. These results imply that the reduction of AB oligomer and phosphorylated tau levels in the brain of mice might lead to an improvement in spatial working memory in the Mucuna group. These results also suggest that Mucuna diet may have protective effects on memory function. However, 3 mice were inactive, and they could not be included in the Y-maze test. Further studies preferably with more than 8 mice per group should be in order to confirm the results of the Y-maze test. Other tests, including a passive avoidance test and a novel object recognition test, should also be conducted.

Mucuna-bean powder has been used for the treatment of Parkinson’s disease as the alternative medicine Levodopa (pharmaceutical formulation of L-DOPA) in India. Parkinson’s disease is a progressive neurodegenerative disorder. Aggregated α-synuclein leads to neuronal death and chronical activation of microglia and astroglia, resulting in the production of inflammatory cytokines such as tumor necrosis factor (TNF-α)^44^. Rai et al. observed significant increases in inflammatory parameters including TNF-α, glial fibrillary acidic protein, inducible nitric oxide synthase and intercellular cell adhesion molecule in the substantia nigra pars compacta of parkinsonian mice^45^. Their study revealed that oral administration of Mucuna-bean-aqueous extracts decreases aforementioned inflammatory parameters^45^. AD is also a progressive neurodegenerative disorder and its progression is related to neuroinflammation, which is led by the progressive activation of microglia and astrocyte with consequent overproduction of inflammatory molecules^46^. Xu et al. reported that intraventricular injection of human Aβ oligomers from AD brain induces inflammatory morphology in microglial cells and that the levels of multiple inflammatory cytokines and chemokine ligands are increased in the brain interstitial fluid^47^. Mucuna beans have been shown to possess potent anti-inflammatory properties, and administration of Mucuna beans might be able to reduce the inflammatory parameters in the brain of AD model mice. Therefore, studying functional activations of microglia and astrocyte would be informative to elucidate anti-inflammatory properties of Mucuna beans in AD brain.

As noted above, a high intake of Mucuna beans sometimes causes nausea and vomiting due to the high L-DOPA content. Sixty PD patients were administered 45 g/person/day of Mucuna-bean powder (containing 1.5 g of L-DOPA) for 12 weeks^16^. Side effects of Mucuna bean powder mainly included mild nausea. Additionally, nine PD patients received 30 g of Mucuna bean powder (containing 1 g of L-DOPA) at once, and then side effects were monitored over the next 240 min. Short-lasting vomiting occurred in one patient, and short-lasting nausea occurred in two patients^48^. No significant changes in hematology or biochemical parameters were observed. However, no information is available on tolerability of Mucuna-bean consumption in healthy subjects. Therefore, caution is needed regarding the intake of Mucuna-bean powder used as a pharmacological dose. Furthermore, some polyphenols are reported to have a potential impact on oxidative damage^49^. Recently, Iijima et al*.* investigated the optimum processing conditions to control the L-DOPA content in Mucuna beans^50^. The content of L-DOPA decreased to approximately 25–40% by processing procedures, while 50%–90% of the other soluble components remained^50^. Iijima et al*.* also developed palatable and nutritious foods using Mucuna beans^51–53^. However, further investigations are needed to clarify the preventive effects of Mucuna beans on AD in humans.

To our knowledge, the present study is the first report that revealed preventive effects of Mucuna beans against AD. Administration of Mucuna beans reduced the levels of Aβ oligomers and detergent-insoluble phosphorylated tau, reduced Aβ accumulation and phosphorylated tau accumulation in the brain, and improved memory function in 3 × Tg-AD mice. These results suggest that Mucuna beans are a candidate food expected to have a preventive effect on AD development.

## Supplementary Information

Supplementary Information 1.

Supplementary Information 2.

Supplementary Information 3.

Supplementary Information 4.

Supplementary Information 5.

Supplementary Information 6.

Supplementary Information 7.

Supplementary Information 8.

## Abbreviations

AD: Alzheimer’s disease
Aβ: Amyloid-beta peptides
NFTs: Neurofibrillary tangles
L-DOPA: L-3,4-dihydroxyphenylalanine
DA: Dopamine
NE: Norepinephrine
DOPAC: 3,4-Dihydroxyphenylacetic acid
APPswe: Amyloid precursor protein Swedish
PS1: Presenilin-1
ThT: Thioflavin T
EDTA: Ethylenediaminetetraacetic acid
EGTA: Ethylene glycol tetraacetic acid
TBS: Tris-buffered saline
PBS: Phosphate-buffered saline
PD: Parkinson’s disease
HPLC: High-performance liquid chromatography
APP: Amyloid precursor protein
TNF-α: Tumor necrosis factor
